# Supplementary material for: Proteomic Characterization of Virulence Factors and Related Proteins in Enterococcus Strains from Dairy and Fermented Food Products
Source: Int J Mol Sci. 2022 Sep 19;23(18):10971. doi: 10.3390/ijms231810971 (PMC9503237; doi:10.3390/ijms231810971)
Supplement: Supplementary file 1 [file ijms-23-10971-s001.zip › Supplemental Data 1.pdf]

International Journal of
Molecular Sciences

MDPI

Article

Proteomic Characterisation of Virulence Factors and Related Proteins in *Enterococcus* Strains from Dairy and Fermented Food Products

Ana G. Abril [1,2], Marcos Quintela-Baluja [3], Tomás G. Villa [1], Pilar Calo-Mata [3], Jorge Barros-Velázquez [3] and M. Carrera [2,*]

1 Department of Microbiology and Parasitology, Faculty of Pharmacy, University of Santiago de Compostela, 15898 Santiago de Compostela, Spain
2 Department of Food Technology, Spanish National Research Council (CSIC), Marine Research Institute (IIM), 36208 Vigo, Spain
3 Department of Analytical Chemistry, Nutrition and Food Science, Food Technology Division, School of Veterinary Sciences, University of Santiago de Compostela, Campus Lugo, 27002, Lugo, Spain
* Correspondence: mcarrera@iim@csic.es

1. Supplemental Data 1

Table S1. Peptides corresponding to bacterial resistance to antibiotics and other toxic substances, identified in the *Enterococcus* strains analyzed.

**Function**	**Protein**	**Sequence**	**Strain**
Antibiotic resistance	TetR family transcriptional regulator	IETTSEMINQK	F1
	TetR family transcriptional regulator	EEVM*DAVIM*RVVEK	F15
	TetR family transcriptional regulator	DVLEVIYAVLNAETIK	F2
	N-acetyltransferase domain-containing protein	SLGYARQTVAKLTADLLAEGK	F7
	PadR domain-containing protein	DLMDGFYKGDE	F3
	GNAT family acetyltransferase	GLGRKM*LGDGMTK	F1
	GNAT family N-acetyltransferase	KAKEMNVSQIDLHVFAHNK	F3
	GNAT family acetyltransferase	QEVDHEDVYKVYNQFAKENHGALQR	F13
	Cass2 domain-containing protein	TYKSDVEHYRK	F15
	MarR family transcriptional regulator	M*NEHDIGMLIKQLYDAKEK	F16
	MarR family transcriptional regulator	M*ENQM*NEQFMK	F5
	Penicillin-binding protein2B	M*NNAYELNTVFIK	F10
	Penicillin-binding protein t	EITENALGTITSAFAPGSVVK	F9
	Penicillin-binding protein	VDKKDDSTYSFSYK	F3
	Daunorubicin resistance protein DrrC	DVSTRIPQGVM*TVISGPAGSGK	F7
	OmpR/PhoB-type domain-containing protein	EVTKILSM*IDQKDHLLIR	F7

	PASTA domain-containing protein	KETTPKQTAPEPASVNVSK	F9
	vanY D-Ala-D-Ala carboxypeptidase	LDKRVVGEYQK	F3
	vanY D-Ala-D-Ala carboxypeptidase	NGVSEEDAIAEVK	F1
	vanY D-Ala-D-Ala carboxypeptidase	IDNDFSVVTNPK	F1
	vanY D-Ala-D-Ala carboxypeptidase	SVAAQQEVFSTNVNSLMSSEGLSEADAIAKTK	F9
Additional resistances and tolerances	Cold shock-like protein CspD	GFGFITTEGGEDIFVHFSAIQGDGFK	F2
	Cold-shock protein (CspA)	TLEEGQSVSFDVEDSDRGPQAVNVEKN	F1
	General stress protein	LVNSDDVTSGINTEVGK	F1
	CsbD-like protein	EGFEDLKDKFSK	F15
	YitT family protein	MAPVLMAVTGR	F2
	Tellurite resistance protein TelA	TQDDLIETIQETLR	F13
	MerR family transcriptional regulator	KLQFYTAKITAQQDSFGWTDFEEAER	F5
	MerR family transcriptional regulator	IVQLQKALNKPK	F15
	SugE protein	DKQSFVLLM*LTFGSSFLFLFLAM*K	F3
	Chaperone protein DnaK	AAVEANDIELMKEKR	F5
	Chaperone protein DnaK	DAEANAEADKLR	F3

M* methionine oxidation; C* carbamidomethylation of Cys.

Table S2. Peptides corresponding to antibacterial compounds and proteins involved in antibacterial production, identified in the *Enterococcus* strains analyzed.

**Function**	**Protein**	**Sequence**	**Strain**
Antimicrobial compounds	Lantibiotic biosynthesis protein	VEDADVEDDC*LC*HGNYGNK	F13
	Type 2 lantipeptide synthetase LanM	FLSILDYPYYLMDQNHYELILEKNLSKK	F11
	LanM family lanthionine synthetase	YINDLKNQNSNK	F5
	Thiopeptide-type bacteriocin biosynthesis domain protein	DSTIKNLESIM*RSLLSNVEYTGYSR	F13
	Bacteriocin Protein	M*VEAAAQRGYIYKK	F2
	Radical SAM additional 4Fe4S-binding SPASM domain-containing protein	KVVFTGGEIGLTHDSLK	F1

M* methionine oxidation; C* carbamidomethylation of Cys.

Table S3. Peptides corresponding to bacterial toxicity, identified in the *Enterococcus* strains analyzed.

**Function**	**Protein**	**Sequence**	**Strain**
Toxin	Type II toxin-antitoxin system PemK/MazF family toxin	VNVPKQGDILLLNAAPR	F1
	Type II toxin-antitoxin system RelE/ParE family toxin	KDGTSEFEEFLNSIPEK	F3
	Addiction module toxin RelE O	ETEVNGM*LVAQRLK	F5

	Toxin-antitoxin system, antitoxin component, AbrB family	SRLQGNSVVVTLPADHGK	F13
	Toxin PIN	KVEKENFLDR	F14
	Exfoliative toxin A/B	KHFLKIIPIPIC*GM*ILGLASLGNLFK	F15
	Prevent-host-death family antitoxin (Phd antitoxin)	STEATTEELKC*GNDHSDYR	F15
	LXG domain-containing protein	ISSGGKLTKDGLK	F5
	LXG domain-containing protein	SLESVSDDLLDSVK	F13

M* methionine oxidation; C* carbamidomethylation of Cys.

Table S4. Peptides corresponding to proteins involved in host colonization and immune evasion, identified in the *Enterococcus* strains analyzed.

**Function**	**Protein**	**Sequence**	**Strain**
Colonization and immune evasion factors	Internalin	EVEEAKEEVDEPTTGVEGSKAEVK	F5
	Sortase	TVDDVTQSIHIQNDAK	F5
	LPXTG cell wall anchor domain-containing protein	DAQNQLSNGAK	F3
	LPXTG cell wall anchor domain-containing protein	AEVGFYAPTNGESDKNDTVSNEGTISPNTNTQGK	F3
	LPXTG cell wall anchor domain-containing protein	MVGM*LMLVLMIPTVC*HAQETETQTIR	F3
	LPXTG cell wall anchor domain-containing protein	LTSAYPAQRNARNVIK	F5
	LPXTG cell wall anchor domain-containing protein	TGETANIALSVLGSLMVLGSGIIFKK	F13
	LPXTG cell wall anchor domain-containing protein	IVTM*PIILTIPK	F14
	LPXTG cell wall anchor domain-containing protein	FTLPDKVTIGLEEKDAVYLIVETK	F15
	LPXTG cell wall anchor domain-containing protein	MHTEEIETANWAEIK	F1
	LPXTG cell wall anchor domain-containing protein	ENLLLDSKM*R	F2
	LPXTG cell wall anchor domain-containing protein	DPDENLAAGQTITIPKGNETLYAVWKK	F2
	LPXTG cell wall anchor domain-containing protein	NTNSKQSSDSKASFNHPSTFFNNNK	F13
	LPXTG cell wall anchor domain-containing protein	VELYTYDAQTK	F14

	LPXTG cell wall anchor domain-containing protein	ETMEFLFEFLVDGC*KLAGKGLSFSEWLQAK	F16
	Adhesin BspA	EM*FKEM*SNLTSLDVSSFDTSK	F3
	Adhesin BspA	GASETLSATVSPVDATDK	F2
	Adhesin BspA	TIPISMNGVVTPGTSSGK	F5
	Adhesin BspA	ADYC*TAC*GALKEYAPNFVENGITNKEC*K	F9
	Fibronectin/fibrinogen-binding protein	EKVFEILSM*TSEINGK	F15
	Collagen-binding protein	GFKITLIGTYQSNM*TK	F16
	Ig domain-containing protein	MNKTIATLTVGATETLSATVSPETATDKSVK	F2
	DUF4097 domain-containing protein	GDLLIQQM*DAAM*LEITGTNNEIELRK	F1
	Cell surface protein	LAASSSEDLNIEIAM*PK	F1
	Flagellar hook-associated protein 2	NFDQVASLLGGEDGLAAK	F5
	Fn3_like domain-containing protein	NDGEIAGKESVLVFVRLLGGAVIQR	F15
	Fimbrial isopeptide formation D2 domain-containing protein	NITATFNVKIGR	F3
	Fimbrial isopeptide formation D2 domain-containing protein	KLADIPSIGDRSLIR	F5
	Fimbrial isopeptide formation D2 domain-containing protein	VHVYPKNELPTNDVKFIK	F9
	Fimbrial isopeptide formation D2 domain-containing protein	GFKNEANVDNGHTVDK	F1
	SpaA domain-containing protein	TAGGDEDKAK	F16
	Endopeptidase NlpC/P60 family protein	ALIDTLGSLPASQDPAPEAR	F13
	Endopeptidase NlpC/P60 family protein	ADLNRQKAEAEAEQAR	F1
	M20/M25/M40 family peptidase	SLEDGKM*HAC*GHDAHTAM*LLTAAR	F11
	M20 peptidase	VGESGLSVTIGKAGK	F15
	Peptidase_M78	IIFGAGDSIKVLK	F2
	Peptidase M28	LVTEVVARLDNEALAEIK	F2
	Peptidase T	TIVEIIKENAKFQK	F2
	DD-transpeptidase	IAGSALHNITNRGGLQGGSTLTQQLIK	F3
	Peptidase	GISMNKQSSSTPLGGLGRGSDK	F12
	Dipeptidase PepV	GSSDDKGPTMAAYYGLK	F13
	Dipeptidase PepV	GSSDDKGPTM*AAYYGLK	F16
	C-terminal processing peptidase	IQNENNLPATGALDDK	F5
	Endopeptidase La	MDFKGVYVM*SVEK	F5
	Peptidase_S8	M*SDSLNQYYFAFGM*GMVLYVLSHFVKKSR	F5
	Dipeptidyl aminopeptidase	ILGGGMSPEDFLK	F5
	Peptidase U32	SIHYVSTVSNVYKAAVDSYM*EDPENYVC*K	F15
	Peptidase S74	QAGEQAQAAAEQASSDAQAAK	F13

	Peptidase, M24 family	KVGLIGWKMFTAK	F13
	Proline dipeptidase	FEALNGFLPGTDFSLDITPVIQK	F5
	Oligoendopeptidase PepF/M3 family protein	LSTYKLHGITDFLQKPLEYNRLQK	F16
	Peptidase C51	EGIAAILGNYSVESGINPK	F16
	Peptidase_S9 domain-containing protein	ILGGGMSPEDFIK	F5
	ImmA/IrrE family metallo-endopeptidase	LKSLM*AYPINALSERK	F15
	ImmA/IrrE family metallo-endopeptidase	TEIVLELLRSNK	F13
	Isoaspartyl dipeptidase	ETLDIDTVIAKGEIM*VQEK	F2
	Isoaspartyl dipeptidase	GGTIDFTASEDPDFWEK	F3
	Signal peptidase I	YIGYFISFMK	F7
	Signal peptidase I	NVLLAILALFSLWFLFNIR	F5
	Signal peptidase I	DSNDSLPKEIQWIEK	F1
	Immune inhibitor A	LDNPATGTPAATGPVK	F2
	ClpA protease	TEVTVQLSKALGIELLR	F11
	ClpC protease	M*GVNETSNASQGTGSRR	F5
	Zinc protease	DPEINEANLNLLK	F15
	Capsular polysaccharide biosynthesis protein CpsC	KRWLLILATTLAGFALAAGVTFFLITPK	F13
	Capsular polysaccharide biosynthesis protein CpsC	MEEMVSLGEMFILLKKR	F16
	N-acetylmuramoyl-L-alanine amidase	AFGDQKKVNLETK	F2
	LysM domain protein	ILKVLAIGITVGGM*ALAIHTEK	F9
	LysM domain protein	KKILVGALVALFFMPTAVLAAK	F13
	Hemolysin III family channel protein	SKKFTHVIWHLFVILGALLM*FFAVYLY	F16
	Autolysin modifier protein	M*NIGSGASVVANNNGTAR	F1
	Mga domain-containing protein	YDGKIWLLNDNNKR	F5
	Mga domain-containing protein	LLQM*NERLIFQYC*MLLRVIFIK	F9
	Mga domain-containing protein	KYDLIITNVSKPNAYRSVK	F4
	Mga domain-containing protein	AYPENEREIIISGILLSSQRAR	F9
	Mga domain-containing protein	QVIDWSWELHYC*EKTVIQK	F1
	Mga domain-containing protein	SEFSKEVNQLYSPLIQELR	F1
	Mga domain-containing protein	MNFFSILDKNTQTQLTILENIYISK	F1
	Mga domain-containing protein	KAVYPLENGIALATVSPK	F16
	Mga domain-containing protein	FNIIIAFIDHLK	F13
	Mga domain-containing protein	DIHPEHESIMLRAGKINLENISATLDEYR	F7
	Mga domain-containing protein	QLIFPKTNSSFVEQLKQLR	F13
	Toxin secretion/phage lysis holin	KVM*ILFVVAVSVK	F4

	Type VII secretion protein EssC	QIENSFDDVNKALRANIPAGMTGTR	F5
	Type VII secretion effector	IAQAVFLASSNLQSVAK	F11
	Type VII secretion protein EssC	DQVLNSLYQILK	F13
	LysR family transcriptional regulator	IAIGGGETQAFSFLAARLNELQQK	F15
	LytR_cpsA_psr domain-containing protein	LIIVFLVLLMGIVSAGTAYAIR	F3
	LytR_cpsA_psr domain-containing protein	IDFTLDGIHVAKGKQTLDGEK	F13
	LytTR family transcriptional regulator	YQISRFAETQMAGINIIM*KNGLTDYVSR	F3
	HTH-type transcriptional regulator KdgR	FLNGRYGNM*SQETK	F1
	ArpU family transcriptional regulator	MLLFPKIDRK	F2
	Competence protein ComEA helix-hairpin-helix repeat region	LIEEQGKLTEELAKSIQK	F3
	Control of competence regulator ComK, YlbF/YmcA	ALQEAYSNVKANEEAHALFK	F5
	Regulatory protein YlbF	M*IAVIVNEELFQLEDQC*LRVAEM*IKK	F9
	Spore coat protein	MENVLC*C*DQIKC*LVGETVKVNLR	F2
	Sporulation protein YjcZ	MGLGGNGGGEAGQAGK	F7
	Restriction endonuclease type IV, Mrr	NKSLYFERMQM*IDM*IR	F13
	SfeI restriction endonuclease	IVMGEVYVIPVYEYDDQAM*INNQVKFKSR	F12
	Type-2 restriction enzyme	VYANVEAM*KIELNILNSLIGSQNVEK	F9
	O-antigen ligase	MLVWQAIYLLVVFQIGK	F7
	Methyl-accepting chemotaxis protein (MCP) signalling domain	STESKLKSMAELPAM*K	F14
	N-acetylglucosamine-6-phosphate deacetylase	M*GATTPADILGEKKIGK	F1
	Glycosyl/glycerophosphate transferases involved in teichoic acid biosynthesis TagF/TagB/EpsJ/RodC	FVQVVEAGEHTVQNM*LK	F9

M* methionine oxidation; C* carbamidomethylation of Cys.

Table S5. Peptides corresponding to transporters associated to virulence factors, identified in the *Enterococcus* strains analyzed.

**Function**	**Protein**	**Sequence**	**Strain**
ABC transporters	Copper ABC transporter permease	YANNTLLMDGFTGIKYNISK	F2
	Copper ABC transporter permease	APTPM*QYLVILLLLLVSGEAIINTR	F11
	Ferrichrome transport ATP-binding protein FhuC	DTGEIYLDHNEVKAWKSNELAK	F13

	Cobalt ABC transporter permease	TKISEWIAALK	F1
	Lantibiotic protection ABC transporter ATP-binding subunit	ERLGIGLALVGNPK	F13
	Multidrug ABC transporter ATP-binding protein	AGEITTANLRR	F3
	Multidrug ABC transporter ATP-binding protein	SNMQWIWR	F2
	Multidrug ABC transporter ATP-binding protein	DADKILYM*QDGDIK	F3
	Spermidine/putrescine ABC transporter-binding protein	EVMGLSLNSLGYSLNSKNNQELR	F5
	Glycine betaine ABC transporter	EMQDELLELQAKVKK	F1
	Glycine betaine/L-proline ABC transporter ATP-binding protein	RPLIDFLDENVMVIGK	F5
	Peptide ABC transporter substrate-binding protein	AMRMADSQEDAK	F1
	Peptide ABC transporter peptide-binding protein	LDEVKTNVVK	F2
	Peptide ABC transporter substrate-binding protein	WKTLQKADQLVAEEAPLVPLYQLTEAR	F5
	Peptide ABC transporter substrate-binding protein	TDGLSIGYLSFNVK	F7
	Peptide ABC transporter ATP-binding protein	M*KKVLLNVQGLK	F13
	His/Glu/Gln/Arg/opine family amino ABC transporter, permease, 3-TM region	IPDLIMNLKNNK	F1
	Amino acid ABC transporter amino acid-binding/permease	IM*VPSFVNQFVITLK	F1
	Amino acid ABC transporter amino acid-binding/permease	DTTIISAIGVVELLQTGK	F1
	Branched-chain amino acid ABC transporter substrate-binding proteinç	FTAGDKDYQAM*LTKVK	F2
	Amino acid ABC transporter ATP-binding protein	ALAMKPDVMLFDEPTSALDPEMVGEVLGVMQR	F5
	Amino acid ABC transporter ATP-binding protein	ASYTEKAVALAK	F15
	Amino acid ABC transporter substrate-binding proteinç	VGTSAEFAPFEFHTLIDGKDK	F16
	Excinuclease ABC subunit A	LALDNGAKHNPK	F2

	Sulfate ABC transporter ATP-binding protein	LPTGNFIPNTDYQAM*YDDATREVKISGILR	F2
	C4-dicarboxylate ABC transporter	DMETYFGQDENK	F4
	Multiple sugar ABC transporter substrate-binding protein	AITVPLKSTGQAQMANFVVSNTSK	F5
	Sugar ABC transporter permease	NTALFAFTVVPISLFLSLGIAWVIFEKVK	F7
	Sugar ABC transporter	ALGDNMGVAAYPTIDFGSGAKQMKAFLGVK	F9
	Carbohydrate ABC transporter substrate-binding protein	DGGKTETITFINHKTDWETNGK	F14
	Sugar ABC transporter permease	STIIIM*LIM*KVGSILTTGFDQIYLM*TNQLNR	F15
	Sugar ABC transporter permease	VGYASAASMVLFFILLLLGLAQMKLGGGKND V	F16
	ABC transporter domain-containing protein	M*RIEVDAISYAYDKNK	F1
	ABC transporter-like protein	GRTTLVIAHRLSTIVDADK	F2
	ABC transporter permease	RSALTM*FGIVIGIAAVIAILSIGRAFER	F2
	ABC transporter permease	GKVATVISGVAVLALIAAATLGAGQSSAK	F2
	ABC transporter domain-containing protein	M*GMFQRMSANDWQK	F2
	ABC transporter domain-containing protein	NLSGGQRQRVALAR	F2
	ABC transporter ATP-binding protein	IM*ALNSSISYM*LMR	F4
	Basic membrane protein (BMP) family ABC transporter substrate-binding protein	GVGTAVQDIANR	F2
	ABC transporter ATP-binding protein	KYVIDDLNLAIK	F2
	ABC transporter domain-containing protein	FGKQEVLKGIDLDVGK	F1
	ABC transporter substrate-binding protein	DM*AEALGIDPESIKEPA	F1
	ABC transporter, ATP-binding protein	NNFLKDNYSLLEGNYPEK	F3
	ABC transporter permease	TFPETFM*EYWPSLIIGLK	F3
	ABC transporter, ATP-binding protein	EKAVNLNNINMEYTLKK	F3
	ABC transporter ATP-binding protein	ALATEPSLLLLDEPFSALDVVTAK	F5
	ABC transporter permease	NMSFKSFYFFTLIGMAILYGTM*WGGR	F5
	ABC transporter domain-containing protein	MIIENINKDMDQRVVLK	F5
	ABC transporter permease	WDSFSTPEFVGIQNFSR	F5

	ABC transporter domain-containing protein	GNAEKFALSMTLAR	F5
	ABC transporter permease	TEENGQLEILKSLGIGAR	F5
	ABC transporter substrate-binding protein	EYNVDGQVTAVNRGEADFAANDFGM*SEERSK	F7
	ABC transporter ATP-binding/permease	SNM*QWIWGYIK	F7
	ABC transporter domain-containing protein	DLAIMIGQEQIVK	F9
	ABC transporter permease	M*LFYENMLMGLLSLVIGIVIGSLLSKGFLK	F9
	ABC transporter domain-containing protein	ILMLAEGRTVAYDTPENLK	F9
	ABC transporter ATP-binding protein	GEIVLTGTVAELK	F10
	ABC transporter permease	NRLPNYRVSFDFTAPK	F11
	ABC transporter domain-containing protein	VVTVVFENFTAELETAIK	F13
	ABC transporter substrate-binding protein	VVALLALSTLVLGLGAC*GGNSAKSKTEDTK	F13
	Heme ABC transporter ATP-binding protein	KAATPKEVVLSIK	F10
	Thiol reductant ABC exporter subunit CydC	EM*IEKLPDGLQTM*VDEAGLR	F14
	ABC transporter domain-containing protein	M*GM*FQRMSANDWQK	F14
	ABC transporter	IGLGATLLLALGGC*GTANSSSAKSDDSK	F14
	ABC transporter ATP-binding protein	SQQLTAFMVTHDMEDAIRYGNRLIM*LHQGK	F14
	ABC transporter permease	KM*VWLM*LLASLIM*PFM*AFLLFNYR	F15
	ABC transporter domain-containing protein	IQEVLQIVSLTNTGKK	F15
	ABC transporter ATPase	GLRYALYGFIAVVVLIMAITLPSGAPLR	F16
	ABC transporter domain-containing protein	MVSMVMAQVVGIDTGSNEPMLK	F16
Other transporters	Major facilitator superfamily (MFS) transporter	LILGIFLFILGFLIGC*LLLK	F2
	Major facilitator superfamily (MFS) transporter	SKKSDSNNSLVK	F7
	Major facilitator superfamily (MFS) transporter	KVM*NESQDAIALKEITQ	F15
	Multidrug resistance MFS transporter	ENLGLAGSLNSFAR	F2

	Cation diffusion facilitator family transporter	KTKGSTAVIAALFANLLVAVSK	F4
	EamA/RhaT family transporter	FIIAFVVLYGILKLAEKK	F5
	Copper-exporting P-type ATPase	IEKNYGVRGMTC*ASC*SQTVEK	F2
	Copper-transporting ATPase CopB	KVDVIM*M*DK	F2

M* methionine oxidation; C* carbamidomethylation of Cys.

Table S6. Peptides corresponding to other virulence factors, identified in the *Enterococcus* strains analyzed.

**Function**	**Protein**	**Sequence**	**Strain**
Alternative virulence factors	Transposase	EMDSLALANAQLNLDK	F5
	Transposase	EM*DSLALANAQLNLDK	F15
	Transposase	LVEMSTPLDIAIPKFK	F5
	Tnp_DDE superfamily	M*TIINATQYLKQLLSSSELNRIGK	F15
	Tnp_DDE superfamily	MTIINATQYLKQLLSSSELNRIGK	F16
	Tnp_DDE superfamily	M*FLLIFLLK	F2
	IS4 family transposase	RAFSLDKLIM*DAGYK	F5
	Transposase InsI for insertion sequence element IS30C	SRFLLAGRILK	F2
	IS30 family transposase	KDGLPKEM*DFNQVDQSFVYAVASLR	F14
	IS4 family transposase	QTIERIFSDEK	F13
	IS6 family transposase	LIRVYGQPRSIVTDK	F13
	Conjugative transposon protein	KVPM*IKVGTHK	F5
	Conjugal transfer protein TraG	M*AINNEDVTDFKAQRNK	F1
	Mutator family transposase	QTVSKEAIYIAI	F3
	Mutator family transposase	WIQVFDELK	F15
	Mutator family transposase	EEAM*DQISFMIDK	F2
	Integrase core domain protein	IESYYHQNIKIVK	F5
	Integrase core domain protein	GYPYHNASLESWHGHLK	F9
	Tyrosine-type recombinase/integrase	AWKFQAYLGINPETGKSVK	F9
	Recombinase family protein	EGRPKKFNQQQINLAM*NLLEDHSYK	F9
	Tyrosine-type recombinase/integrase	C*PFTNEPPC*LSC*NNGNPC*K	F7
	Enterococcus faecalis plasmid pPD1 bacI	ETQKIADQLNSSGSQHNAGSYEFYDMAAMLK	F14
	Putative plasmid replication protein	TVLWSGVLHLADK	F2
	PrgI family protein	M*AVEVKVPKDIK	F1
	Pheromone response system RNA-binding regulator PrgU	M*EAVVEREAKGM*K	F3

	Regulatory protein RecX	GFSGDITNLVLANLELEIDEDLEKEALKK	F13
	Regulatory protein RecX	TNVRLSDKGPK	F2
	CRISPR-associated endonuclease Cas9	QSVSINQKLLKEK	F3
	CRISPR-associated endonuclease Cas10	KYRAFFGDNSVNGYAGYIEGHATQEDFYK	F9
	CRISPR-associated endoribonuclease Cas2	MSYRYMRM*ILM*FDM*PTETAEER	F16
	YqaJ domain-containing protein	KKGIGGSDVGTILGLNK	F1
	Luciferase family oxidoreductase, group 1	EAFPEDHLYSK	F1
Phage proteins	HK97 family phage major capsid protein (*Enterococcus* sp. 8G7_MSG3316)	AAAGTLTFAKNTIVSELAGVM*KK	F3
	Phage capsid protein (*Enterococcus casseliflavus)*	EAEERENEAAR	F1
	Phage head protein gp7 (*Enterococcus* phage phiFL2A and *Enterococcus faecalis*)	M*DEIMAYVDK	F3
	Minor head protein (*Enterococcus casseliflavus*)	MNKEKIM*TDSFLGFDYR	F5
	Capsid protein (*Enterococcus villorum*)	EKDLEIAIEEAKTEEEQQVVEDEVNK	F5
	Long-tail fiber protein gp35 (*Enterococcus saccharolyticus*)	M*LLNISEKLM*AK	F13
	Phage tail protein (*Enterococcus faecium*)	LFMDDISQAMGEGADINLEF	F5
	Phage tail protein (*Enterococcus faecium*)	EMGITITNGRIEQNMDMFSESTRK	F7
	Phage tail tape measure protein (*Enterococcus* avium)	M*SSIGSTLTM*SVTAPIAAGFGAATK	F2
	Phage tail tape measure protein TP901 (*Enterococcus durans*)	GLAIASAIFGSM*SLLTGGLVVALGALIGALVVAYTK	F2
	Phage tail tape measure protein, TP901 family, core region (*Enterococcus faecalis*)	WGTDVGKATDGALTK	F3
	Phage tail tape measure protein (*Enterococcus avium* and *Enterococcus* devriesei)	GVQYM*NEYQDGVNTNAEK	F3
	Phage tail sheath (*Enterococcus* phage 163 , *Enterococcus* phage vB_OCPT_Ben, *Enterococcus* phage	IVQDVTTYNDK	F3

	EfsSzw-1 , *Enterococcus* phage EFP01, *Enterococcus* phage EFDG1, *Enterococcus* phage PEf771, *Enterococcus* phage EfV12-phi1, *Enterococcus* phage 156)		
	Phage tail tape measure protein (*Enterococcus faecium*, *Enterococcus hirae*)	IDMNNLMEASDVLSDIGDKLTELGK	F1
	Phage tail tape measure protein, TP901 family, core region (*Enterococcus hermanniensis*)	EQTSEM*ITALGNLSEK	F1
	Phage tail tape measure protein, TP901 family (*Enterococcus faecalis*)	KTPTSPFSDPK	F5
	Phage tail tape measure protein (*Enterococcus hirae*)	VM*IEGIRSEQGQEQM*NHMVMDLQR	F5
	Tail length tape-measure protein (*Enterococcus* phage PBEF129, *Enterococcus* phage EFLK1, *Enterococcus* phage phiEF17H , *Enterococcus* phage phiEF24C, *Enterococcus* phage vB_EfaM_Ef2.3, *Enterococcus* phage vB_EfaH_EF1TV, *Enterococcus* phage ECP3, *Enterococcus* phage 156, *Enterococcus* phage phiM1EF22)	TRAENNAEESANLSIYSK	F5
	HK97 gp10 family phage protein (*Enterococcus faecalis*)	RSETPSIKDDGMTGEVEAMADYSAYVEYGTR	F1
	Phage portal protein, HK97 family (*Enterococcus faecalis*)	EDGKDIKGAK	F5
	Putative phage portal protein, SPP1 family (*Enterococcus faecalis*)	DLFLGNVSAKIKAPK	F3
	Phage portal protein *Enterococcus* gallinarum	ITAHVWKFAMAVNTILC*GNSYSR	F3
	Phage portal protein (*Enterococcus plantarum*)	AGSQLHIM*KGITPEITDEGIK	F5
	Phage/plasmid primase, P4 family domain protein *Enterococcus faecium*	DM*IVTSEVLAKELEIK	F16
	PBSX family phage terminase, large subunit (*Enterococcus faecalis*)	VNKSPM*KITYK	F15
	Phage terminase large subunit	QFNTAYIEIAKKQGK	F3

	(*Enterococcus cecorum* , *Enterococcus faecium*)		
	Phage integrase (*Lactococcus garvieae*)	RTISRESLMLYR	F2
	Phage_integrase domain-containing protein (*Enterococcus malodoratus*)	IANGAVEAM*VGK	F3
	Site-specific recombinase, phage integrase family (*Enterococcus faecalis*)	AGEMIALTWSDIDFYNQTVR	F3
	Phage integrase family protein (*Enterococcus faecium*)	VHQLTRVEIEAYLSELNMM*GIKPSTITGR	F5
	Cro/CI family transcriptional regulator (*Enterococcus faecium*)	NLNIGNNIKNIR	F13
	HTH cro/C1-type domain-containing protein (*Enterococcus* sp.)	M*LDSNVNMSYGGENLTEDEK	F14
	HTH cro/C1-type domain-containing protein (*Enterococcus rotai*)	DYKALFLHC*AQNM*HDENK	F15
	HTH cro/C1-type domain-containing protein (*Enterococcus saccharolyticus* subsp. *saccharolyticus*)	FIVSEDEKDFFNTVISGHYQLKK	F16
	HTH cro/C1-type domain-containing protein (*Enterococcus moraviensis*)	KKEYEEC*LYYLK	F1
	Cro/Cl family transcriptional regulator (*Enterococcus rotai, Enterococcus* haemoperoxidus, *Enterococcus caccae*)	ETSINVADFQNK	F13
	XRE family transcriptional regulator (*Enterococcus thailandicus*)	EGTKMSLGDKLK	F10
	XRE family transcriptional regulator (*Enterococcus termitis*)	DNIKQFLVDAK	F2
	XRE family transcriptional regulator (*Enterococcus faecalis*)	MSQDLAIEVRAALIRAGK	F16
	BppU family phage baseplate upper protein (*Enterococcus faecium*)	LGDLKPVEGLTDVWSLTITPTK	F2

	BppU_N domain-containing protein (*Enterococcus faecalis*)	DEIPDLSGGIITFEGEPAGGK	F11
	phage infection protein YhgE/Pip domain-containing protein (*Enterococcus asini*)	RIAKSPVAVFLIVALM*ILPSLYAWFNIK	F13
	Uncharacterized protein (*Enterococcus* phage vB_EfaM_Ef2.3, *Enterococcus* phage phiEF24C, *Enterococcus* phage EFLK1, *Enterococcus* phage vB_EfaM_Ef2.1, *Enterococcus* phage 156)	YAQSMWKLANTIQKSGISR	F16
	Uncharacterized protein (*Enterococcus* phage LY0323, *Enterococcus* phage IME-EF4, *Enterococcus* phage vB_EfaS_AL2, *Enterococcus* phage Ec-ZZ2)	GNSKSFDPM*RAGQHDVVVIESTEK	F15
	Uncharacterized protein (*Enterococcus hirae*)	KTM*TAILAEMTKK	F5
	Phage protein (*Enterococcus* phage 156)	KTNEEYLK	F3
	Uncharacterized protein gp54 (*Enterococcus* phage phiFL3A, *Enterococcus casseliflavus*)	LDEGIM*GLFVK	F2
	Membrane protein (*Enterococcus* phage heks)	DQHAANM*M*LDAM*IK	F3
	Phage-associated protein, HI1409 family (*Enterococcus faecalis*)	NDFM*LGNGK	F1

M* methionine oxidation; C* carbamidomethylation of Cys.

Table S7. Peptides corresponding to bacteriophage proteins, identified in the *Enterococcus* strains analyzed, that were determined as homologues to proteins found in different *Enterococcus* bacteriophages.

**Bacteriophage protein**	**Bacteriophage**	**Peptide sequence**	Strain
Phage head protein gp7	*Enterococcus* phage phiFL2A and *Enterococcus faecalis*	M*DEIMAYVDK	F3
Phage tail sheath	*Enterococcus* phage 163, *Enterococcus* phage vB_OCPT_Ben, *Enterococcus* phage EfsSzw-1 , *Enterococcus* phage EFP01, *Enterococcus* phage EFDG1, *Enterococcus* phage	IVQDVTTYNDK	F3

	PEf771, *Enterococcus* phage EfV12-phi1, *Enterococcus* phage 156		
Tail length tape-measure protein	*Enterococcus* phage PBEF129, *Enterococcus* phage EFLK1, *Enterococcus* phage phiEF17H, *Enterococcus* phage phiEF24C, *Enterococcus* phage vB_EfaM_Ef2.3, *Enterococcus* phage vB_EfaH_EF1TV, *Enterococcus* phage ECP3, *Enterococcus* phage 156, *Enterococcus* phage phiM1EF22	TRAENNAEESANLSIYSK	F5
Uncharacterized protein	*Enterococcus* phage vB_EfaM_Ef2.3, *Enterococcus* phage phiEF24C, *Enterococcus* phage EFLK1, *Enterococcus* phage vB_EfaM_Ef2.1, *Enterococcus* phage 156	YAQSMWKLANTIQKSGISR	F16
Uncharacterized protein	*Enterococcus* phage LY0323, *Enterococcus* phage IME-EF4, *Enterococcus* phage vB_EfaS_AL2, *Enterococcus* phage Ec-ZZ2	GNSKSFDPM*RAGQHDVVVIESTEK	F15
Phage protein	Phage protein	KTNEEYLK	F3
Uncharacterized protein gp54	*Enterococcus* phage phiFL3A, *Enterococcus casseliflavus*	LDEGIM*GLFVK	F2
Membrane protein	*Enterococcus* phage heks	DQHAANM*M*LDAM*IK	F3

M* methionine oxidation; C* carbamidomethylation of Cys.
